# A Pilot Head-to-Head Comparison of Two Bisulfite Conversion Kits for NGS-Based DNA Methylation Analysis of Liquid Biopsy Samples from Colorectal Cancer

**DOI:** 10.3390/cancers18193148

**Published:** 2026-09-29

**Authors:** Omayma Mazouji, Abdelhak Ouhajjou, Roberto Incitti, Chakib Nejjari, Menter David, Morris Van Karlyle, Raghav Kanwal Pratap Singh, Scott Kopetz, Hicham Mansour

**Affiliations:** 1 GES-LCM2E, FSA Mohamed First University, Nador 62000, Morocco; mazouji.omayma@ump.ac.ma; 2 Gastrointestinal Medical Oncology, MD Anderson Cancer Center, University of Texas, Houston, TX 77030, USA; 3 Al-Azhar Oncology Center, Rabat 10000, Morocco; 4 Euromed Reseach Center, Euromed University of Fes, Fes 30070, Morocco; 5 Faculty of Medicine, Pharmacy and Dentistry, Sidi Mohamed Ben Abdellah University, Fes 30070, Morocco

**Keywords:** Zymo, EpiTect, bisulfite conversion, DNA methylation, cfDNA, WGBS

## Abstract

Given the important role of early detection in improving cancer outcomes, epigenetic alterations, particularly DNA methylation, have emerged as powerful biomarkers for cancer screening and diagnosis. However, methylation profiling of cfDNA (circulating free DNA) presents particular technical challenges because cfDNA is typically present at very low concentrations and is highly fragmented. Bisulfite conversion, although essential for distinguishing methylated from unmethylated cytosines, can further compromise DNA integrity through chemical-induced loss of DNA during purification steps. Inadequate conversion or excessive DNA loss may consequently reduce library complexity, increase DNA duplication rates, and negatively affect mapping efficiency and downstream sequencing quality. Therefore, optimizing bisulfite conversion is particularly important for reliable methylation profiling of low-input cfDNA. The effectiveness of these diagnostic approaches relies on the accurate detection of DNA methylation patterns, which is fundamentally dependent on the efficiency of bisulfite conversion of genomic DNA. This pilot study aims to compare the performance of two widely used bisulfite conversion kits, namely the EpiTect Bisulfite (EpiTect) Kit (Qiagen, Hilden, Germany) and the EZ DNA Methylation (Zymo) Kit (Zymo Research, Irvine, CA, USA), in the context of plasma-derived cfDNA methylation profiling using five samples, including two plasma samples from metastatic colorectal cancer (mCRC) patients collected at the Department of Gastrointestinal Oncology at The University of Texas MD Anderson Cancer Center, in Houston, TX, USA, and two plasma samples from healthy donors (controls) purchased from Innovative Research (Novi, MI, USA), along with one control DNA sample (Corriel NA12878-N88). Our objective was to evaluate and compare their conversion efficiency, mapping efficiency (percentage of mapped and unmapped reads), duplication rate, and distribution of methylated cytosines in CpG, CHG, and CHH contexts. Our results indicate that samples processed using the Zymo kit consistently outperformed those treated with the EpiTect kit across multiple performance metrics. The Zymo-treated samples yielded a higher concentration of cfDNA following bisulfite conversion (125.34 nM vs. 45.60 nM in samples processed with the Epitect kit). Additionally, the Zymo kit achieved a higher average bisulfite conversion efficiency (99.3% vs. 95.7%), as well as a significantly higher average mapping rate (94.86% vs. 72.4%), with fewer unmapped reads and lower duplication rates. Head-to-head comparison of Bismark methylation calls across the same reference regions showed high concordance between the two protocols. The SEPT9 region was strongly hypermethylated with both protocols (100% with EpiTect and approximately 94% with Zymo), whereas the BMP3 region was predominantly unmethylated (approximately 4% with EpiTect and 7% with Zymo). These findings suggest that the Zymo kit demonstrated improved performance under the conditions evaluated for cfDNA methylation analysis compared to the EpiTect protocol. Further investigation using a larger number of samples is needed to validate our findings.

## Simple Summary

DNA methylation is a promising biomarker for the early detection and diagnosis of cancer using circulating cell-free DNA (cfDNA). Reliable methylation analysis depends on efficient bisulfite conversion, a key step in preparing DNA for sequencing. In this pilot study, we compared two commonly used bisulfite conversion kits: the EpiTect Bisulfite Kit and the EZ DNA Methylation Kit. The EZ DNA Methylation Kit showed superior performance, producing higher cfDNA yields, greater bisulfite conversion efficiency, improved mapping rates, and lower duplication rates. These findings suggest that this kit provides a more reliable approach for cfDNA methylation profiling and may improve the accuracy of liquid biopsy-based cancer detection.

## 1. Introduction

Unlike genetics, which involves changes in gene function caused by alterations in the DNA sequence, epigenetics refers to changes in gene activity that occur without modifying the DNA sequence [1]. DNA methylation, a major epigenetic phenomenon, is a covalent modification in which a methyl group (CH_3_), donated by S-adenosylmethionine (SAM-CH_3_), is added to the fifth carbon of the cytosine pyrimidine ring, producing 5-methylcytosine and converting StableAM-CH_3_ into S-adenosylhomocysteine (SAH) [2]. This process predominantly occurs at CpG dinucleotides, especially within regions of high CpG density known as CpG islands. These islands are often located in gene promoters and first exons, where they can regulate gene transcription. In contrast, the rest of the genome typically contains low CpG density. DNA methylation is a reversible process mediated by DNA methyltransferases (DNMTs). There are three main DNMTs with specific functions: DNMT1 maintains methylation patterns during DNA replication, while DNMT3A and DNMT3B are responsible for de novo methylation by adding new methyl groups to previously unmethylated cytosines [3,4]. In addition, DNA demethylation can occur passively during DNA replication or actively through specific enzymes known as Ten-Eleven Translocation (TET) enzymes, which oxidize methylated cytosine, followed by the base excision repair mechanism [5].

Consequently, DNA methylation affects gene expression by either attracting proteins that repress transcription or by preventing the binding of transcription factors to DNA [6]. Methylation in gene promoter regions is typically associated with transcriptional silencing, whereas methylation within gene bodies is often linked to active gene transcription [7].

Given the important role of early detection in improving cancer outcomes, epigenetic alterations have emerged as powerful biomarkers for cancer screening, diagnosis, prognosis, and treatment prediction [8]. Among these, aberrant DNA methylation, especially the early occurrence of tumor suppressor gene silencing during tumorigenesis, plays a crucial role in tumor initiation and progression, making it a valuable indicator for early detection.

Recent advancements in the analysis of plasma-derived cell-free DNA have enabled non-invasive epigenetic profiling through liquid biopsy, offering a powerful tool for detecting cancer-specific methylation patterns at early stages [7]. According to the literature, numerous methylated genes have been identified as promising biomarkers for colorectal cancer (CRC) detection. Several of these biomarkers have already been incorporated into commercially available kits for early CRC screening, such as the Epi proColon, Cologuard, and ColoSure tests [9]. These tests target biomarkers such as SEPT9, NDRG4, and BMP3, some of which are included in FDA-approved assays for clinical use [10,11,12,13,14].

The success of these diagnostic tools relies on the accurate detection of DNA methylation, which in turn depends on efficient bisulfite conversion of genomic DNA. Sodium bisulfite treatment has revolutionized the field of DNA methylation analysis by allowing the differentiation between methylated and unmethylated cytosines. This chemical approach converts unmethylated cytosine into uracil, while leaving methylated cytosines unchanged. These patterns can then be analyzed across specific genomic regions or the entire genome using next-generation sequencing-based methods, polymerase chain reaction (PCR)-based technologies and other approaches [15]. Recent studies have further highlighted the growing potential of cfDNA methylation sequencing for liquid biopsy applications, while also emphasizing important methodological challenges associated with low cfDNA abundance, DNA fragmentation, DNA conversion efficiency, library preparation, sequencing coverage, and analytical reproducibility [16,17]. In particular, bisulfite conversion remains widely used for methylation sequencing but can cause substantial DNA loss, which is particularly challenging when working with the limited and fragmented DNA available from plasma. Recent comparisons of bisulfite-based and enzymatic methylation sequencing approaches have demonstrated that different workflows can produce substantial differences in mapping efficiency, genomic coverage, conversion efficiency, reproducibility, and library quality, highlighting the importance of optimizing the pre-sequencing workflow for cfDNA applications [17].

Moreover, the clinical translation of cfDNA methylation assays requires greater standardization of pre-analytical and analytical procedures. Recent work has emphasized variability introduced by plasma processing, cfDNA extraction, DNA input, conversion chemistry, library preparation, and sequencing, while efforts toward standardized reference materials and quality-control procedures are still ongoing [18]. Importantly, the choice of bisulfite conversion kit may influence DNA recovery and conversion efficiency, and these effects may subsequently affect downstream library complexity, mapping performance, duplication rates, and methylation measurements. Therefore, although previous studies have compared commercially available bisulfite conversion kits using purified DNA or conventional samples, their performance may not necessarily translate directly to low-input, fragmented cfDNA obtained from cancer patient plasma. This gap provides the rationale for the present pilot study. To the best of our knowledge, few studies have directly compared the performance of the EpiTect Bisulfite Kit (Qiagen, Hilden, Germany) and the EZ DNA Methylation Kit (Zymo Research, Irvine, CA, USA) using whole-genome bisulfite sequencing-based quality metrics in plasma-derived cfDNA from CRC patients and healthy controls. Rather than simply comparing bisulfite conversion percentages, our study evaluates multiple downstream indicators, including post-conversion DNA recovery, bisulfite conversion efficiency, mapping efficiency, unmapped reads, duplication rate, and the distribution of methylated cytosines across the CpG, CHG, and CHH genomic sequence contexts. This comprehensive comparison is particularly relevant because a conversion protocol that achieves high chemical conversion efficiency may nevertheless result in substantial DNA loss or poor sequencing performance. Therefore, evaluating the complete workflow under the same experimental conditions may provide practical information for selecting an appropriate bisulfite conversion protocol for low-input cfDNA methylation profiling and contribute to ongoing efforts toward greater standardization of liquid biopsy methylation workflows.

In this pilot study, we compare the performance of two widely used bisulfite conversion kits, namely the EpiTect Bisulfite Kit (Qiagen, Hilden, Germany) and the EZ DNA Methylation Kit (Zymo Research, Irvine, CA, USA), in the context of plasma-derived cfDNA methylation profiling. Our objective is to evaluate and compare their conversion efficiency, mapping efficiency (percentage of mapped and unmapped reads), duplication rate, and the distribution of methylated cytosines in CpG, CHG, and CHH genomic sequence contexts. These metrics are critical for assessing the quality and suitability of each kit for downstream whole-genome bisulfite sequencing (WGBS) analysis. Given the fragmented and low-abundance nature of plasma-derived cfDNA, the choice of an optimal bisulfite conversion protocol is essential for enabling accurate detection of methylation biomarkers in non-invasive cancer diagnostics.

## 2. Materials and Methods

### 2.1. Pilot Test Samples

To assess the performance of two bisulfite conversion kits, the EpiTect Bisulfite Kit (Qiagen, Hilden, Germany) and the EZ DNA Methylation Kit (Zymo Research, Irvine, CA, USA), a pilot test was conducted using five samples: two plasma samples from patients with mCRC, two plasma samples from healthy donors, and one control DNA sample (Coriell NA12878-N88). The clinical plasma samples were obtained from the Department of Gastrointestinal Oncology at The University of Texas MD Anderson Cancer Center, Houston, Texas, USA. Blood samples were obtained after written informed consent from all participants, in accordance with institutional ethical standards and approved study protocols by the appropriate ethics committee. The study was conducted in accordance with the Declaration of Helsinki and approved by the Institutional Review Board of The University of Texas MD Anderson Cancer Center (protocol code: IRB00000121; date of approval: 6 May 2026).

Both mCRC patients had advanced-stage disease and subsequently died. The first patient was a 37-year-old male who received one line of treatment consisting of FOLFOX plus bevacizumab (FOLFOX + B); his blood sample was collected on 7 May 2012. The second patient was a 60-year-old female who received four lines of treatment: FOLFOX plus bevacizumab, 5-fluorouracil (5-FU) plus leucovorin plus bevacizumab, FOLFIRI plus bevacizumab, and cetuximab plus irinotecan; her blood sample was collected on 19 January 2011. Both samples were stored at −80 °C and processed on 20 February 2025, corresponding to storage durations of approximately 12 years and 9 months and 14 years and 1 month, respectively.

For the healthy donor controls, one female donor (58 years old) and one male donor (58 years old) were included. The female donor sample was collected on 24 July 2024, whereas the male donor sample was collected in May 2024. Both samples were processed on 20 February 2025, corresponding to approximately 7 months and 9 months of storage, respectively. These plasma samples were purchased from Innovative Research (Novi, MI, USA). Each donor sample was tested for standard FDA-required viral markers and was negative for HBsAg, HCV, HIV-1, HIV-2, HIV-1 antigen or NAT, ALT, West Nile Virus NAT, Zika NAT, and syphilis using FDA-approved methods. The control DNA sample, Coriell NA12878-N88, is to the well-known unmethylated bacteriophage lambda (λ) genome, which was included as a reference control for the bisulfite conversion and downstream DNA methylation analyses.

For the clinical samples, 20 mL of blood was collected in Streck Cell-Free DNA BCT tubes. Plasma was separated within the first few hours following blood collection. Plasma processing consisted of two centrifugation steps: the first at 1000 rpm for 10 min, followed by a second centrifugation at 6000 rpm for 10 min. The resulting plasma samples were stored at −80 °C until DNA isolation and subsequent assays. The storage duration varied according to the interval between plasma collection and DNA isolation/analysis. This pilot comparison was designed to evaluate the performance and efficiency of the two bisulfite conversion kits under the available clinical and control sample conditions for downstream DNA methylation analysis.

### 2.2. Isolation and Quantification of Circulating Cell-Free DNA

CfDNA was extracted from 5 mL of plasma using the QIAamp Circulating Nucleic Acid Kit (Qiagen, Hilden, Germany), according to the manufacturer’s instructions. The cfDNA extraction, quantification, and subsequent experimental procedures were performed at Admera Health Laboratory (South Plainfield, NJ, USA). The isolated cfDNA was either immediately processed for bisulfite conversion or stored at −20 °C for future use. A total of five cfDNA samples were extracted and subsequently evaluated using both bisulfite conversion protocols, allowing paired comparison of the two kits using the same starting biological material.

### 2.3. DNA Bisulfite Conversion and Quantitative Analysis

We conducted a comparative assessment of two commercially available bisulfite conversion kits, namely the EpiTect Bisulfite Kit (Qiagen, Hilden, Germany) and the EZ DNA Methylation Kit (Zymo Research, Irvine, CA, USA) to evaluate their performance. For bisulfite conversion using the EZ DNA Methylation Kit (Zymo Research, Irvine, CA, USA), which accepts a DNA input ranging from 500 pg to 2 µg, 20 µL of extracted cfDNA was combined with 130 µL of freshly prepared CT Conversion Reagent, resulting in a total reaction volume of 150 µL. The samples were incubated at 98 °C for 10 min followed by 64 °C for 2.5 h. The converted DNA was subsequently purified using the supplied Zymo-Spin IC spin-column procedure. Briefly, 600 µL of M-Binding Buffer was added to the column, followed by loading of the converted DNA. The column was washed with 100 µL of M-Wash Buffer and subsequently with 200 µL of M-Desulphonation Buffer (Zymo Research, Irvine, CA, USA), which was incubated for 15–20 min at room temperature. Two additional washes were performed using 200 µL of M-Wash Buffer each. The converted DNA was finally eluted in 20 µL of elution buffer, of which 10 µL was used for library preparation. The same bisulfite conversion procedure was applied to each of the five cfDNA samples. For the EpiTect Bisulfite Kit, the manufacturer’s protocol was followed using a DNA input within the recommended range of 1 ng–2 µg, with a maximum DNA volume of 20 µL. The bisulfite conversion reaction was brought to a total volume of 140 µL and subjected to thermal cycling at 95 °C for 5 min, 60 °C for 25 min, 95 °C for 5 min, 60 °C for 85 min, 95 °C for 5 min, followed by 60 °C for 175 min. The converted DNA was purified using the EpiTect spin-column procedure and eluted in 20 µL of Buffer EB. For each of the five cfDNA samples, the extracted cfDNA was processed independently using both conversion kits, such that each biological sample generated one Zymo-treated library and one EpiTect-treated library. This paired experimental design ensured that the two conversion protocols were evaluated using the same biological starting material. After bisulfite conversion, the treated DNA was quantified using the Qubit™ dsDNA High Sensitivity Assay Kit (Invitrogen, Waltham, MA, USA) on a Qubit^®^ 3.0 Fluorometer (Thermo Fisher Scientific, Waltham, MA, USA). The resulting bisulfite-converted cfDNA was either processed immediately for library preparation or stored at −20 °C for later use.

### 2.4. Library Preparation

Library preparation of the bisulfite-treated DNA was performed using the xGen™ Methyl-Seq Library Preparation Kit (Integrated DNA Technologies, Coralville, IA, USA), according to the manufacturer’s protocol. The DNA input amount ranged from 7 to 400 ng for both kits, depending on the amount of DNA available for each sample. PCR amplification was performed using 14 cycles. Libraries were indexed using 8-nucleotide (8-nt) CDI indexes. Libraries generated from both Zymo- and EpiTect-treated samples were prepared in the same experimental batch using the same library-preparation workflow to minimize potential batch-to-batch variation. Following library preparation, the concentration of each library was measured using the Qubit™ dsDNA High Sensitivity Assay Kit (Invitrogen, Waltham, MA, USA) on a Qubit^®^ 3.0 Fluorometer (Thermo Fisher Scientific, Waltham, MA, USA).

### 2.5. Quantitative and Qualitative Assessment of Bisulfite- Converted cfDNA Libraries

The prepared libraries underwent both quantitative and qualitative quality control assessments. Quantitative analysis was carried out using the QuantStudio™ 5 Real-Time PCR System (Applied Biosystems, Waltham, MA, USA) to accurately quantify the concentration of DNA in the prepared libraries, while qualitative evaluation was performed using the TapeStation High Sensitivity D1000 Assay (Agilent Technologies, Santa Clara, CA, USA) to assess the size distribution and integrity of library fragments. The final library size was approximately 340 bp, corresponding to an estimated DNA insert size of approximately 220 bp after accounting for adapter and index sequences. No technical replicates were included; each sample–kit combination was represented by a single library.

### 2.6. Whole Genome Bisulfite Sequencing

The prepared libraries were subjected to whole-genome bisulfite sequencing using the NovaSeq X Plus platform (Illumina, San Diego, CA, USA) with a 10B flow cell, enabling high-throughput sequencing. Sequencing was performed using 150 bp paired-end reads (PE150), with 150 bp generated in each sequencing direction. Each sample generated approximately 60 million paired-end (PE) reads, corresponding to 60 million reads per direction, then 120 million single reads were generated, in total. For each of the five biological samples, the Zymo- and EpiTect-derived libraries were normalized, equal amounts pooled and sequenced in the same sequencing run and on the same flow-cell lane, thereby minimizing potential variation attributable to sequencing batch or lane effects. Sequencing was performed according to standard protocols, ensuring consistency and high data quality across all samples.

### 2.7. Preliminary Evaluation of WGBS Output and Statistical Analysis

The raw FASTQ files generated from whole-genome bisulfite sequencing were processed using a standardized analysis workflow on the Galaxy server (https://usegalaxy.eu, accessed on 1 June 2025). Initial quality assessment of the raw sequencing reads was performed using FastQC to evaluate base quality and sequence content. Following this, for the four plasma samples, the reads were aligned to the GRCh38 human reference genome using the Bismark Mapper, while the Coriell controls were mapped to the well-known unmethylated bacteriophage lambda (λ) genome reference. In addition to the standard quality control metrics, bisulfite conversion efficiency was assessed to evaluate the reliability of the conversion process, which is critical for accurate DNA methylation analysis. For each cytosine context (CpG, CHG, and CHH), the methylation percentage was calculated as: % Methylated = [Methylated Cytosines/(Methylated Cytosines + Unmethylated Cytosines)] × 100. Methylated cytosines were defined as cytosines retaining a methylation mark, whereas unmethylated cytosines were defined as cytosines without a methylation mark. Non-CpG methylation was therefore calculated separately for the CHG and CHH contexts using the same formula. Bisulfite conversion efficiency was calculated using the formula: Efficiency = 100 – (% non-CpG methylation), where the lower value between CHH and CHG methylation levels was used as a proxy for unconverted cytosines. For statistical analysis, comparisons between the Zymo and EpiTect conversion protocols were performed using measurements obtained from five paired samples processed under both conditions. Because the same samples were analyzed using both conversion protocols, the measurements were treated as paired observations. The normality of the paired differences was assessed using the Shapiro–Wilk test. The Shapiro–Wilk test indicated a significant deviation from normality only for CpG methylation (*p* < 0.05). Accordingly, two-tailed paired Student’s *t*-tests were performed for the normally distributed comparisons, including qPCR concentration, conversion efficiency, mapping percentage, duplication rate, CHG/CHH methylation, and Phred quality score > 30. These analyses were performed using Srplot, corresponding to a two-tailed, paired Student’s *t*-test (TTEST function in Microsoft Excel type = 1, tails = 2). Statistical significance was defined as *p* < 0.05. For the non-normally distributed CpG methylation data, the non-parametric paired Wilcoxon signed-rank test was applied. To assess the practical significance of the observed differences across all comparisons, paired Cohen’s *d* effect sizes were calculated.

### 2.8. Comparative Analysis of Methylation Calls in Reference Regions

To assess the consistency of methylation measurements between the two bisulfite conversion protocols, Bismark methylation calls were compared head-to-head using the same genomic coordinates for both the Zymo and EpiTect libraries. Two reference regions, SEPT9 (chr17: 75,369,000–75,369,499) and BMP3 (chr4: 81,030,950–81,031,449), were selected for comparison. Bismark-generated cytosine methylation calls were extracted for all covered CpG sites within each predefined genomic window. For each CpG site, methylated and unmethylated reads were identified from the Bismark methylation output, and the methylation level was calculated as the proportion of methylated reads among the total methylated and unmethylated reads: methylation (%) = [Methylated Cytosines/(Methylated Cytosines + Unmethylated Cytosines)] × 100. The per-cytosine methylation calls were then summarized across each reference region to obtain an overall methylation level for each sample and conversion protocol. The same genomic coordinates and calculation procedure were applied to the Zymo- and EpiTect-derived libraries, allowing direct comparison of methylation levels and assessment of variability between the two bisulfite conversion workflows.

## 3. Results

### 3.1. Quantitative Analysis of cfDNA Libraries

Before bisulfite conversion and library preparation, the amount of extracted DNA available for conversion was assessed for each sample. The mean DNA input for bisulfite conversion was 122.12 ± 169.37 ng for both the EpiTect and Zymo protocols, as the same extracted DNA inputs were used for the paired samples for bisulfite conversion followed by sequencing library preparation (Supplementary Materials, Table S1).

The quantity of prepared cfDNA libraries was measured using a qPCR approach to compare the library concentrations generated by two different conversion kits: EZ DNA Methylation Kit and EpiTect Bisulfite Kit (Figure 1).

For the five samples analyzed, the Zymo kit yielded significantly higher library concentrations than the EpiTect kit. The mean library concentration was 125.34 nM for the Zymo kit, compared with 45.61 nM for the EpiTect kit. The mean paired difference was 79.73 nM (95% CI: 31.79–127.68 nM), with a statistically significant difference between the two protocols (*p* = 0.0099, paired *t*-test). These findings indicate that the Zymo protocol produced higher post-conversion library concentrations than the EpiTect protocol, suggesting improved library yield and potentially greater availability of DNA for downstream sequencing applications Consistent with the qPCR findings, Qubit measurements also showed higher mean library concentrations for the Zymo protocol (12.50 ± 1.36 ng/µL) compared with the EpiTect protocol (6.75 ± 4.45 ng/µL), with a significant paired difference of 5.75 ng/µL (95% CI: 0.19–11.31 ng/µL; *p* = 0.0453). These Qubit results are provided in the Supplementary Materials (Table S1).

The intensity of the bands generated by tapstation reflects the relative concentration of the prepared library molecules across the different size ranges, providing an additional check of the DNA library concentration. Notably, samples processed with the Zymo kit exhibited higher TapeStation concentrations (4153.33 ± 1827.63 pg/µL) compared with the EpiTect kit (1309.67 ± 511.65 pg/µL), with a mean paired difference of 2843.67 pg/µL. However, this difference did not reach conventional statistical significance (95% CI: −480.92 to 6168.26 pg/µL; paired *p* = 0.0665). Therefore, the results indicate a trend toward higher library concentration with the Zymo protocol, rather than a statistically significant difference in library concentration between the two protocols (Supplementary Materials, Table S2).

### 3.2. Evaluation of cfDNA Librairies Quality Using TapeStation Technique

The quality of prepared libraries was assessed using TapeStation analysis (Figure 2). The electrophoresis profile displays the size distribution of the final sequencing libraries generated from plasma-derived cfDNA. This DNA library includes the original DNA inserts together with the adapter and barcode/index sequences introduced during library preparation. The predominant library size was approximately 340 bp, corresponding to an estimated DNA insert size of approximately 220 bp after accounting for the adapter and barcode/index sequences. DNA integrity was evaluated based on the fragment-size distribution and the percentage of integrated area obtained from the TapeStation electropherograms. Across the samples, the predominant fragments were concentrated within the approximately 325–415 bp range, with most samples showing a single dominant fragment population accounting for 100% of the integrated area. For the EpiTect control samples, 76.3% of the integrated area corresponded to the main 325 bp fragment, while an additional 23.7% was detected at approximately 436 bp. The CRC samples processed with EpiTect and Zymo, as well as the Corriel samples processed with both kits, showed 100% integrated area associated with the predominant library peak, indicating a relatively homogeneous fragment distribution and no substantial evidence of low-size fragmented products in the analyzed libraries (Supplementary Materials, Table S3).

### 3.3. Comparative Assessment of Conversion Kits

To assess and optimize bisulfite conversion efficiency for plasma-derived cfDNA, a comparative evaluation was conducted using Zymo and the EpiTect kits. Conversion performance was assessed using several quality control metrics, including percentage of conversion efficiency, percentage of sequenced bases that have a Phred quality score of 30 or higher, percentages of mapped, unmapped reads, and duplication rate, and the distribution of methylated cytosines in CpG, CHG, and CHH contexts.

We assessed differences between the Zymo and EpiTect conversion protocols using five paired replicates, comparing key molecular and sequencing metrics. The normality of the data differences was assessed using the Shapiro–Wilk test, and the only comparison for which the Shapiro–Wilk test detected a significant deviance from a normal distribution (*p* < 0.05) was “% CpG methylation”. Consequently, a two-tailed paired Student’s *t*-test was also performed for the comparisons, namely “qPCR concentration”, “conversion efficiency”, “mapping percentages”, “duplication rates”, and “CHG/CHH methylation” and “Phred quality score >30”. For all except the latter the *t*-test revealed statistically significant differences (*p* < 0.05) between Zymo and Epictet. Conversely, a non-parametric paired Wilcoxon signed-rank test was applied to the methylated CpG data, which yielded a marginal statistical trend (*p* = 0.0625). To assess the practical significance of these shifts across all comparisons, we calculated paired Cohen’s d effect sizes, consistently obtaining a strong (d > 1.0) effect size (Supplementary Materials, Table S2).

• **Percentage** **of conversion efficiency**

As summarized in Figure 3, the Zymo Kit demonstrated higher bisulfite conversion efficiency compared with the EpiTect Kit across the five paired samples. The Zymo kit achieved a mean conversion efficiency of 99.3%, with all five samples showing a conversion efficiency of 99.3%. In contrast, the EpiTect kit showed a lower mean conversion efficiency of 95.7%, with individual values ranging from 93.3% to 98.2%. The mean paired difference between the two protocols was 3.60 percentage points (95% CI: 1.14–6.06), indicating a statistically significant difference (paired *t*-test, *p* = 0.0154). These findings indicate that the Zymo kit achieved significantly higher bisulfite conversion efficiency than the EpiTect kit under the conditions evasluated, which may support more reliable cfDNA methylation profiling.

• **Percentage of sequenced bases that have a Phred quality score of 30 or higher**

To assess sequencing performance, we examined the percentage of sequenced bases with a Phred quality score of 30 or higher across all analyzed samples (Figure 4). Both kits consistently yielded Q30 values with mean percentages of 95.26 ± 0.39% for the Zymo kit and 95.10 ± 0.55% for the EpiTect kit. The mean paired difference between the two kits was 0.16 percentage points (95% CI: −0.18 to 0.50), indicating only a small difference in Q30 performance. This difference was not statistically significant (paired *p* = 0.2560). Thus, both conversion kits demonstrated comparable sequencing quality based on the proportion of bases achieving a Q30 score or higher.

• **Percentages of mapped, unmapped reads, and duplication rate**

In terms of sequencing alignment, a comparative analysis of mapping metrics revealed performance differences between the Zymo and EpiTect bisulfite conversion kits (Figure 5).

The Zymo-treated samples showed a higher mean **mapping rate** of 94.86 ± 0.70%, compared with 72.40 ± 17.03% for the EpiTect-treated samples (Figure 5A). The mean paired difference in mapping rate was 22.46 percentage points (95% CI: 0.69–44.23), and this difference was statistically significant (paired *t*-test, *p* = 0.0457). Consistent with the higher mapping rate, the percentage of unmapped reads was substantially lower for the Zymo kit (5.14 ± 0.70%) than for the EpiTect kit (27.62 ± 17.04%) (Figure 5B). The mean paired difference was −22.48 percentage points (95% CI: −44.26 to −0.70), indicating a statistically significant difference between the two conversion protocols (paired *t*-test, *p* = 0.0457).

**Duplication rates** were also lower in the Zymo-treated samples, with a mean of 7.98 ± 1.97%, compared with 15.66 ± 2.66% for the EpiTect-treated samples (Figure 5C). The mean paired difference was −7.68 percentage points (95% CI: −11.20 to −4.16), and the difference was statistically significant (paired *t*-test, *p* = 0.0037).

Regarding genome-wide sequencing coverage, the Zymo non-directional libraries achieved an average coverage of approximately 2.3×, with approximately 71% of the genome covered at ≥1×. In comparison, the EpiTect directional libraries achieved an average coverage of approximately 1.4×, with approximately 57% of the genome covered at ≥1×.

Overall, these results indicate that the Zymo workflow was associated with a higher mapping rate, fewer unmapped reads, and a lower duplication rate than the EpiTect workflow. However, these downstream sequencing and library metrics can be influenced by multiple stages of the experimental workflow and should therefore not be attributed solely to differences in bisulfite conversion chemistry.

• **Distribution of methylated cytosines in CpG, CHG, and CHH contexts**

Regarding the distribution of methylated cytosines across CpG, CHG, and CHH contexts, the two conversion kits showed similar CpG methylation levels (Figure 6). The mean CpG methylation was 74.17 ± 12.64% for Zymo and 75.47 ± 12.35% for EpiTect, with a mean paired difference of −1.31 percentage points (95% CI: −2.75–0.14). This difference was not statistically significant according to the paired Wilcoxon signed-rank test (*p* = 0.0625). In contrast, substantially higher non-CpG methylation levels were observed with the EpiTect kit. CHG methylation averaged 0.674 ± 0.021% for Zymo and 4.282 ± 1.964% for EpiTect, with a mean paired difference of −3.61 percentage points (95% CI: −6.05 to −1.16; *p* = 0.0149). Similarly, CHH methylation was 0.752 ± 0.041% for Zymo and 4.404 ± 2.092% for EpiTect, corresponding to a mean paired difference of −3.65 percentage points (95% CI: −6.24 to −1.06; *p* = 0.0173). Overall, while CpG methylation profiles were comparable between the two kits, Zymo produced significantly lower levels of CHG and CHH methylation. As non-CpG methylation can serve as an indicator of incomplete bisulfite conversion, these lower CHG and CHH methylation levels are consistent with the higher conversion efficiency observed for the Zymo kit.

### 3.4. Head-to-Head Comparison of Methylation Levels

To directly assess methylation variability between the two bisulfite conversion protocols, Bismark methylation calls were compared head-to-head across the same genomic regions in the Zymo- and EpiTect-treated cfDNA libraries. For each reference region, per-cytosine methylation calls were summarized across the corresponding genomic window by counting methylated and unmethylated reads at each covered CpG site. The comparison demonstrated a high degree of concordance between the two protocols and preserved the expected methylation patterns of both selected regions of the well-known genes (Figure 7). In the selected SEPT9 region (chr17: 75,369,000–75,369,499), which represents a hypermethylated target, methylation was detected at 100% in the EpiTect-treated libraries and approximately 94% in the Zymo-treated libraries. Thus, both protocols consistently identified SEPT9 as strongly hypermethylated, with only a modest difference of approximately 6 percentage points between the two workflows. In contrast, the selected region of the *BMP3* gene (chr4: 81,030,950–81,031,449), representing an essentially unmethylated region, showed very low methylation levels with both protocols, at approximately 4% for EpiTect and 7% for Zymo. The difference between the two workflows was therefore small (approximately 3 percentage points), and both approaches consistently classified BMP3 as predominantly unmethylated. Overall, the head-to-head comparison of Bismark methylation calls across identical genomic coordinates showed that the two conversion protocols produced highly concordant methylation profiles, with the same hypermethylated and unmethylated patterns observed in both reference regions. These findings indicate that, despite differences in sequencing metrics, the choice of bisulfite conversion protocol did not substantially alter the methylation status detected at these selected genomic regions. Importantly, this downstream methylation assessment was restricted to two selected genomic regions and was intended as a preliminary technical evaluation rather than a comprehensive assessment of downstream biological or clinical performance.

## 4. Discussion

The detection of DNA methylation biomarkers in cfDNA-based blood samples and other bodily fluids holds significant clinical potential for cancer diagnosis and monitoring. However, only a limited number of methylation-based liquid biopsy assays have been successfully applied in routine clinical practice. A major obstacle hindering broader implementation is the low abundance of tumor-derived DNA within the circulating cfDNA pool, which necessitates highly sensitive and efficient analytical methods. Recent studies have further emphasized that the clinical utility of liquid biopsy depends not only on the analytical sensitivity of the assay but also on the efficient recovery and preservation of limited circulating cell-free DNA molecules during sample processing and library preparation [19,20]. In this pilot study, we compared two widely used bisulfite conversion kits, including the EpiTect Bisulfite Kit (Qiagen, Hilden, Germany) and the EZ DNA Methylation Kit (Zymo Research, USA), in the context of plasma-derived cfDNA methylation profiling. We evaluated and compared their conversion efficiency, mapping efficiency (percentage of mapped and unmapped reads), duplication rate, distribution of methylated cytosines in CpG, CHG, and CHH contexts, and measurements of methylation levels of CpG in selected regions of two well-known genes, *SEPT9* and *BMP3*. Our results indicate that samples processed using the Zymo kit showed improved performance across several analytical metrics compared with those converted with the EpiTect kit. The Zymo-treated samples yielded a higher post-conversion library concentration. Additionally, the Zymo kit achieved higher average bisulfite conversion efficiency and exhibited notably higher mapping rates. Although these findings are broadly consistent with previous reports describing favorable performance of Zymo-based kits, the main contribution of the present study is the assessment of whether differences between the two conversion workflows extend beyond conversion efficiency to downstream sequencing and library-quality metrics in plasma-derived cfDNA. Importantly, these findings should be interpreted as an analytical comparison within the conditions of this pilot study rather than as evidence that one kit is universally superior.

Bisulfite conversion is a critical chemical treatment used in most DNA methylation analysis methods and represents an essential step in epigenetic studies. However, this procedure often leads to DNA loss, which can affect downstream analysis. Additionally, incomplete bisulfite conversion is a well-recognized issue, leading to overestimation of methylation levels, thereby skewing the accuracy of the results [21]. Because bisulfite conversion involves harsh chemical treatment, it presents a trade-off between achieving the desired conversion of unmethylated cytosines to uracils and minimizing DNA loss or incorrect conversion of methylated cytosines. Previous research has consistently demonstrated that the bisulfite kits used here generally achieve high conversion rates. Kint and colleagues performed a comprehensive comparison of twelve commercially available bisulfite conversion kits using blood samples. Among these, the EZ DNA Methylation-Gold Kit (Zymo Research) demonstrated the highest conversion efficiency, achieving 99.7%. The EpiTect Bisulfite Kit (Qiagen) ranked fourth among the twelve kits evaluated, achieving 98.3% [22]. Additionally, Hong and Shin performed a comparative analysis of six bisulfite conversion kits. Their results revealed that most of the kits achieved conversion efficiency greater than 99%, with the EZ DNA Methylation-Lightning Kit (Zymo Research) demonstrating the highest efficiency, followed by EpiTect Fast DNA Bisulfite Kit (Qiagen) [21]. Holmes and colleagues also evaluated the performance of nine commercially available bisulfite conversion kits. Based on the data generated, the EZ DNA Methylation-Gold Kit yielded the highest DNA quantities. In addition, the conversion efficiencies were generally high across all kits, ranging from 98.7% for EpiTect Bisulfite Kit to 99.9% for EZ DNA Methylation-Direct Kit. These results further support the superior performance of Zymo-based kits compared to EpiTect kits [23]. Thus, the favorable conversion efficiency and DNA recovery observed with Zymo in the present study largely confirm trends already reported in previous kit-comparison studies rather than representing a novel observation in isolation.

Unlike previous studies that identified the EZ DNA Methylation-Gold™ Kit as the top- performing bisulfite conversion kit, Kresse and collaborators revealed that the EpiTect Plus DNA Bisulfite Kit surpassed the Zymo kit in several key performance metrics. Among the five bisulfite conversion kits evaluated, the EpiTect kit yielded the highest DNA concentrations across a range of input amounts, followed closely by the Premium and Zymo kits. Furthermore, electrophoretic analysis demonstrated that the EpiTect kit produced the longest average DNA fragment sizes. Based on these findings, the EpiTect kit was ranked as the best-performing kit in this study. These results highlight that kit performance may vary depending on experimental conditions and study objectives, emphasizing the need for context-specific validation in DNA methylation studies [24]. This context dependence is important when interpreting the present findings. Rather than establishing a universal ranking between Zymo and EpiTect, our study evaluates their performance under a specific plasma-derived cfDNA sequencing workflow and demonstrates that differences may become apparent when downstream sequencing-quality metrics are considered.

Through comparative analysis of bisulfite conversion kits using sequencing alignment metrics, we identified notable performance differences between the Zymo and EpiTect kits. Samples processed using the Zymo protocol achieved a significantly higher average mapping rate (94.86%) compared to EpiTect (72.4%), with fewer unmapped reads and lower duplication rates indicating superior data quality and overall mapping performance. However, given the very small number of biological samples evaluated, these statistical comparisons should be interpreted cautiously. In the revised analysis, the same five samples processed with both kits were treated as paired observations, and individual sample-level paired comparisons were considered alongside statistical significance. Effect sizes and 95% confidence intervals are also reported where applicable to provide information on the magnitude and precision of the observed differences rather than relying solely on *p*-values. Importantly, the value of this comparison lies not simply in confirming that Zymo can perform favorably, but in demonstrating that the choice of conversion workflow may be associated with differences in downstream sequencing usability, including mapping efficiency, unmapped reads, and duplication rate. These downstream metrics were not the primary focus of the earlier kit-comparison studies cited above and therefore provide an additional perspective on conversion-kit performance. The observed differences in mapping and duplication rates likely reflect the combined influence of DNA recovery, fragment integrity, library preparation, adapter ligation, PCR amplification, sequencing depth, and bioinformatic processing. Thus, the contribution of the bisulfite conversion kit to these downstream differences cannot be isolated from the other steps of the workflow. While both kits showed comparable efficiency in detecting CpG methylation, EpiTect showed slightly higher levels of non-CpG methylation (CHG and CHH), suggesting increased background noise. Because non-CpG methylation, particularly CHG and CHH methylation, is generally expected to be low in mammalian DNA, elevated levels of these methylation contexts following bisulfite treatment may indicate incomplete conversion of unmethylated cytosines. Thus, CHG and CHH methylation levels represent the proportion of methylated cytosines among all informative cytosines within each respective non-CpG context. These residual non-CpG methylation levels were considered as a proxy for unconverted cytosines and were therefore used in the assessment of bisulfite conversion efficiency presented in Figure 3. The slightly higher CHG/CHH methylation observed for EpiTect may consequently reflect a greater proportion of incompletely converted cytosines and a potential increase in background methylation calls. However, this interpretation should be considered cautiously, as low levels of genuine non-CpG methylation may also contribute to the observed CHG and CHH signals. The substantially higher mapping rate observed with the Zymo-treated samples is particularly relevant for plasma-derived DNA because cfDNA is already highly fragmented and present at relatively low concentrations. Recent cfDNA methylome studies have demonstrated that fragmentation characteristics are closely linked to the information obtainable from plasma DNA and that methylation and fragmentation features can be jointly exploited for cancer detection [19,20,25].

The relevance of these quality-control metrics to downstream methylation analysis is important. Post-conversion DNA recovery directly affects the number of DNA molecules available for library construction and sequencing; therefore, greater recovery can increase the number of informative molecules available for methylation calling, which is particularly important for low-input and highly fragmented plasma cfDNA [19,20]. Bisulfite conversion efficiency is also critical because incomplete conversion of unmethylated cytosines can result in their being incorrectly interpreted as methylated cytosines, leading to an overestimation of DNA methylation levels [21]. In this context, non-CpG methylation, particularly CHG and CHH methylation, can be used as an indicator of incomplete conversion in human somatic DNA. Thus, lower residual CHG and CHH methylation is consistent with more complete bisulfite conversion and reduces the potential for false-positive methylation calls [21,22]. Accordingly, the present study extends the conventional assessment of bisulfite conversion efficiency by considering whether conversion-related differences are accompanied by changes in downstream sequencing characteristics and methylation-context profiles.

Mapping rate is relevant because reads that cannot be reliably aligned to the reference genome cannot be confidently assigned to genomic loci and therefore cannot contribute reliably to locus-specific methylation estimates. A higher mapping rate consequently increases the proportion of sequenced molecules that can be used for methylation calling and genomic interpretation [26]. Similarly, duplication rate provides an indication of library complexity. High duplication can occur when a limited number of original DNA molecules are repeatedly amplified during PCR, resulting in multiple sequencing reads originating from the same molecule. Consequently, a high duplication rate can reduce the effective sequencing depth because additional reads do not necessarily represent additional independent DNA molecules. Lower duplication rates therefore generally indicate greater library complexity and a larger proportion of unique molecules contributing to the methylation dataset. Evaluating these metrics alongside conversion efficiency is therefore particularly relevant for low-input plasma cfDNA, where relatively small differences in molecule recovery or library complexity may have a greater downstream impact than would be expected from conversion efficiency alone.

The Q30 metric provides an additional measure of sequencing-base quality. A high proportion of bases with a Phred quality score ≥ 30 indicates a low expected base-calling error rate and supports more reliable downstream read alignment and methylation analysis [27]. Although both kits produced high Q30 values in the present study and the observed difference was small, maintaining high sequencing quality is important because poor-quality bases can impair read alignment and potentially reduce the accuracy of methylation calls. Collectively, these metrics provide complementary information: DNA recovery reflects the availability of molecules for library construction, conversion efficiency and non-CpG methylation reflect the quality of bisulfite conversion, mapping rate reflects the proportion of reads that can be confidently assigned to the reference genome, duplication rate reflects the representation of unique molecules, and Q30 reflects sequencing-base accuracy. The combination of these complementary metrics, rather than conversion efficiency alone, represents an important methodological aspect of the present comparison.

One possible explanation for the higher mapping rate observed with Zymo-treated samples is improved preservation or recovery of DNA during the bisulfite-conversion workflow. Bisulfite treatment can damage DNA and reduce the amount and length of DNA molecules available for subsequent library preparation. Recent methodological work on whole-genome bisulfite sequencing has specifically emphasized that bisulfite-induced DNA damage and loss can limit library complexity, particularly when starting with low amounts of DNA, and that minimizing DNA loss during end repair, adapter ligation, purification, and amplification can improve library quality [28]. Therefore, the higher post-conversion DNA concentration observed with the Zymo protocol in our study may have resulted in a larger pool of intact, amplifiable molecules entering library preparation, potentially contributing to the higher proportion of reads that could be successfully aligned to the reference genome. Although fragment size distributions were obtained and library construction was successful in the present study, this suggested mechanism remains a hypothesis rather than a demonstrated explanation.

The higher library concentration observed with the Zymo protocol was also supported by three independent quantification approaches, each using a different protocol: qPCR, Qubit, and TapeStation. qPCR showed a significantly higher mean library concentration for Zymo than for EpiTect (125.34 ± 22.22 vs. 45.61 ± 31.12 nM; mean paired difference = 79.73 nM; 95% CI: 31.79–127.68; paired *p* = 0.0099). Similarly, Qubit measurements demonstrated a significantly higher concentration for Zymo (12.50 ± 1.36 vs. 6.75 ± 4.45 ng/µL; mean paired difference = 5.75 ng/µL; 95% CI: 0.19–11.31; paired *p* = 0.0453). TapeStation analysis showed the same directional trend, with higher library concentrations for Zymo than EpiTect (4153.33 ± 1827.63 vs. 1309.67 ± 511.65 pg/µL); however, this difference did not reach conventional statistical significance (mean paired difference = 2843.67 pg/µL; 95% CI: −480.92–6168.26; paired *p* = 0.0665). Importantly, the absolute concentrations obtained by these methods should not be directly compared because they are based on different measurement principles and are expressed in different units. Rather, the consistent direction of the differences across all three methods provides complementary evidence supporting the higher library concentration obtained with the Zymo protocol. qPCR estimates amplifiable library molecules, Qubit measures total DNA concentration, and TapeStation provides concentration information together with the size distribution of the final library. The lack of statistical significance for the TapeStation comparison may reflect the greater variability observed with this specific measurement and the limited number of paired samples in this pilot study.

Differences in DNA fragmentation may also contribute to the observed mapping disparity. Plasma cfDNA consists predominantly of short, naturally fragmented DNA molecules, and additional fragmentation or damage during bisulfite treatment can further reduce the proportion of sequencing molecules that retain sufficient information for reliable alignment. Recent studies using cfDNA methylome sequencing have shown that fragment size, methylation, and genomic coverage are interconnected features of plasma DNA and can be simultaneously informative for cancer detection [20,25]. Thus, a conversion workflow that better preserves short cfDNA fragments could plausibly generate a greater proportion of mapped reads.

Another potential contributor is adapter ligation and subsequent library amplification. Bisulfite-converted DNA contains a high proportion of uracil-containing and potentially damaged molecules, making efficient adapter ligation and subsequent amplification important determinants of library complexity. Recent cfDNA sequencing studies have emphasized the importance of minimizing DNA loss during library preparation, particularly when only small amounts of fragmented plasma DNA are available. In addition, PCR amplification can disproportionately represent a limited subset of molecules, increasing duplicate reads and reducing library complexity. The lower duplication rate observed in the Zymo-treated samples may therefore be consistent with greater library complexity or more efficient recovery of distinct DNA molecules. Nevertheless, adapter-ligation efficiency and PCR amplification bias were not directly assessed in our experiment, and therefore these mechanisms cannot be conclusively attributed to the observed difference in mapping rate. Future studies incorporating fragment-size analysis, library yield, adapter-ligated molecule quantification, unique molecular identifiers, and PCR-cycle optimization would be useful for determining which aspects of the workflow account for the difference.

The direct comparison of the two bisulfite conversion kits further demonstrated their similar performance in detecting DNA methylation across the two selected genomic regions. Both the Zymo and EpiTect protocols consistently identified the expected methylation profiles, with high methylation levels in the SEPT9 region and very low methylation levels in the BMP3 region. Although EpiTect showed slightly higher methylation levels than Zymo for SEPT9 (100% versus approximately 94%) and lower levels for BMP3 (approximately 4% versus 7%), these differences were relatively small and did not affect the overall classification of the two regions as hypermethylated and predominantly unmethylated, respectively. Therefore, the results suggest that both conversion protocols are capable of preserving and detecting the expected methylation patterns in these target regions. However, no statistical analysis was performed to formally assess differences between the two kits, as such an analysis would require a larger number of genomic regions and a broader set of CpG sites to provide sufficient statistical power and a more representative assessment of protocol-dependent variability. Importantly, this two-locus analysis was intended as a preliminary descriptive assessment of downstream methylation concordance and does not establish methylome-wide reproducibility, consistency of differential methylation, or sensitivity for biomarker detection. Similarly, these findings should not be interpreted as evidence of general biological or clinical superiority of either conversion protocol. The comparison of only two selected regions is therefore considered descriptive and should be interpreted as an initial, pilot-level assessment of concordance between the two bisulfite conversion workflows rather than a comprehensive evaluation of downstream biological or clinical performance.

Several limitations should be considered when interpreting the present findings. First, this was a small pilot study involving only two colorectal cancer plasma samples, two healthy-control samples, and one Coriell reference DNA sample. Therefore, the sample size is insufficient to establish the statistical robustness, biological variability, or clinical generalizability of the observed differences. Although the same five samples were evaluated using both conversion protocols, allowing paired comparisons, the very small number of paired observations limits statistical power. Consequently, *p*-values should not be interpreted independently of the magnitude and consistency of the observed paired differences. Second, multiple sequencing and quality-control metrics were evaluated simultaneously, including conversion efficiency, Q30, mapping rate, unmapped reads, duplication rate, methylation-context distributions, and measurements of methylation levels of CpG in selected genomic regions. This multiple-metric analysis increases the possibility of type I error when individual statistical tests are considered separately. Given the exploratory and preliminary nature of this pilot study and the very small sample size, the analyses were primarily intended to characterize performance differences rather than to provide definitive hypothesis testing. Multiple-comparison correction was therefore not used as the basis for selecting a single statistically significant outcome; instead, the potential for multiple-testing effects is acknowledged as a limitation, and the results are interpreted collectively together with effect sizes, confidence intervals, and individual sample-level paired comparisons. Future studies with larger sample sizes should incorporate an appropriate multiple-comparison correction and a predefined primary endpoint. Third, the study evaluated only two commercially available bisulfite conversion kits. Therefore, the findings cannot be generalized to other bisulfite-conversion platforms, kit formulations, or alternative methylation-analysis workflows. Differences in DNA input amount, conversion conditions, purification procedures, library-preparation methods, sequencing depth, and bioinformatic pipelines may also influence kit performance. Accordingly, the observed advantage of the Zymo protocol should be considered specific to the experimental and analytical conditions used in this study rather than interpreted as evidence of universal superiority. Fourth, the present study focused on analytical and sequencing-quality metrics and did not include downstream biological analyses such as differential methylation analysis, identification of cancer-associated methylation loci, biomarker discovery, or validation of methylation markers for colorectal cancer detection. Therefore, although the Zymo protocol showed favorable performance for several technical metrics, we cannot determine from the present data whether these differences translate into improved biological interpretation, cancer discrimination, or clinical diagnostic performance. Larger studies incorporating differential methylation analysis, independent validation cohorts, and clinically relevant performance measures will be required to determine whether the observed technical differences have meaningful biological or clinical consequences. Finally, the present study did not directly quantify all factors that may contribute to differences in sequencing performance, including DNA recovery at each processing step, adapter-ligation efficiency, library complexity, PCR amplification bias, and fragment-size distributions of the native cfDNA before library preparation. The TapeStation analysis was performed on the final libraries and therefore reflects library molecules containing both DNA inserts and library-adapter/index sequences rather than the native cfDNA size distribution. Consequently, the mechanisms underlying the observed differences in mapping and duplication rates require further investigation to help clarify these mechanisms.

The biological and analytical relevance of these findings is particularly important for plasma-based methylation analysis. Recent large-scale studies have demonstrated that genome-wide methylation profiling of cfDNA can provide clinically informative cancer-associated signals and that methylation information can be complemented by fragmentation and other genomic features [20,29]. In this context, maximizing the proportion of high-quality, uniquely mappable reads is important because inefficient mapping or excessive duplication reduces the number of informative molecules available for methylation calling and may ultimately limit sensitivity when tumor-derived DNA represents only a small fraction of total plasma cfDNA.

These findings indicate that, under the conditions evaluated in this pilot study, the Zymo kit was associated with improved downstream sequencing performance compared with the EpiTect kit for bisulfite sequencing of plasma-derived cfDNA. However, the principal contribution of the present study is not the confirmation that Zymo can perform favorably, as this has been reported previously, but the extension of kit comparison to downstream sequencing and library-quality characteristics within the same paired plasma-derived DNA samples. To the best of our knowledge, few studies have directly compared the performance of the EpiTect Bisulfite Kit and the EZ DNA Methylation Kit using whole-genome bisulfite sequencing-based quality metrics in plasma-derived cfDNA from colorectal cancer patients and healthy controls. Rather than simply comparing bisulfite conversion percentages, our study evaluates multiple downstream indicators, including post-conversion DNA recovery, bisulfite conversion efficiency, mapping efficiency, unmapped reads, duplication rate, the distribution of methylated cytosines across CpG, CHG, and CHH contexts, and the measurement of methylation levels of CpG in selected regions of two well-known genes. This multidimensional assessment provides a complementary perspective to previous studies. In particular, the paired evaluation of several metrics, ranging from bisulfite DNA treatment to methylation calling, allows the potential downstream consequences of conversion-workflow choice to be considered alongside conventional conversion metrics. Nevertheless, because of the small sample size, limited number of conversion kits, and absence of downstream biological and clinical validation, these findings should be regarded as preliminary and hypothesis-generating. This pilot study provides additional insight into how the choice of bisulfite conversion kit may affect downstream sequencing quality and methylation analysis of plasma-derived cfDNA and provides a basis for larger, adequately powered comparative studies.

## 5. Conclusions

In conclusion, based on the comparative evaluation of two widely used bisulfite conversion kits, in this small pilot evaluation, the Zymo kit demonstrated improved analytical performance. It yielded the highest average DNA concentration, achieved superior conversion efficiency, and exhibited higher mapping rates across all analyzed samples, although the methylation levels in two well-known genomic regions were equivalent between the two kits. These preliminary technical observations highlight the critical importance of selecting an appropriate bisulfite conversion kit, as it can substantially influence the quantity and quality of cfDNA available for downstream analyses. These findings highlight the importance of evaluating bisulfite conversion workflows in the specific context of low-input plasma-derived cfDNA and downstream sequencing applications. Validation in a larger, independent cohort will be necessary to determine whether the observed differences are reproducible and generalizable across samples and experimental conditions.

## Figures and Tables

**Figure 1:**
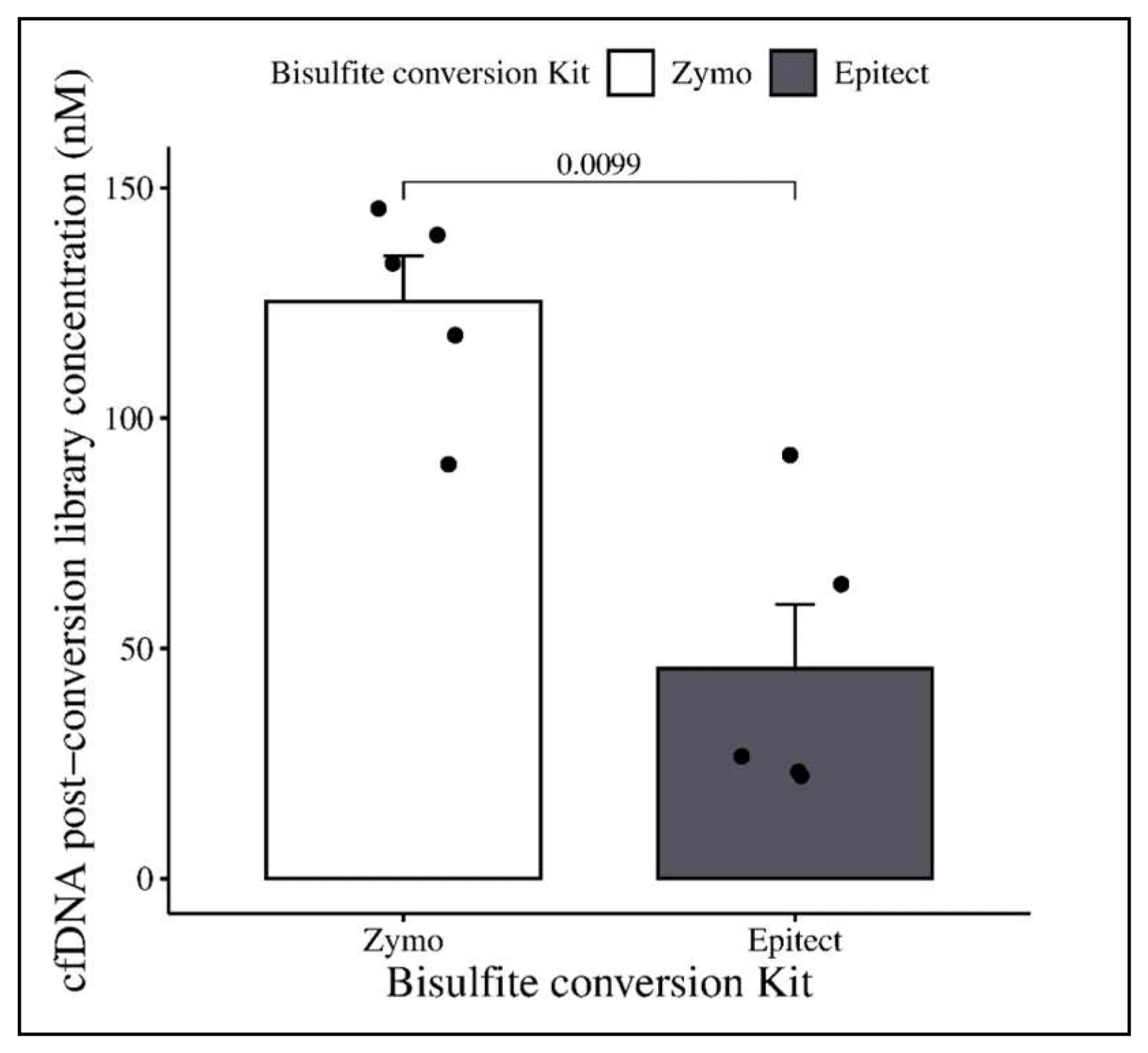
Comparison of post-conversion library concentrations measured by qPCR to evaluate the performance of two conversion kits, Zymo and EpiTect. Data are presented as individual paired observations together with the mean ± SD (*n* = 5). Lines connecting measurements represent the same biological sample processed with the two conversion protocols. The mean library concentration was 125.34 ± 22.71 nM for Zymo and 45.61 ± 30.35 nM for EpiTect. The mean paired difference was 79.73 nM (95% CI: 31.79–127.68 nM). The difference in library concentration between the two conversion protocols was statistically significant (paired *t*-test, *p* = 0.0099).

**Figure 2:**
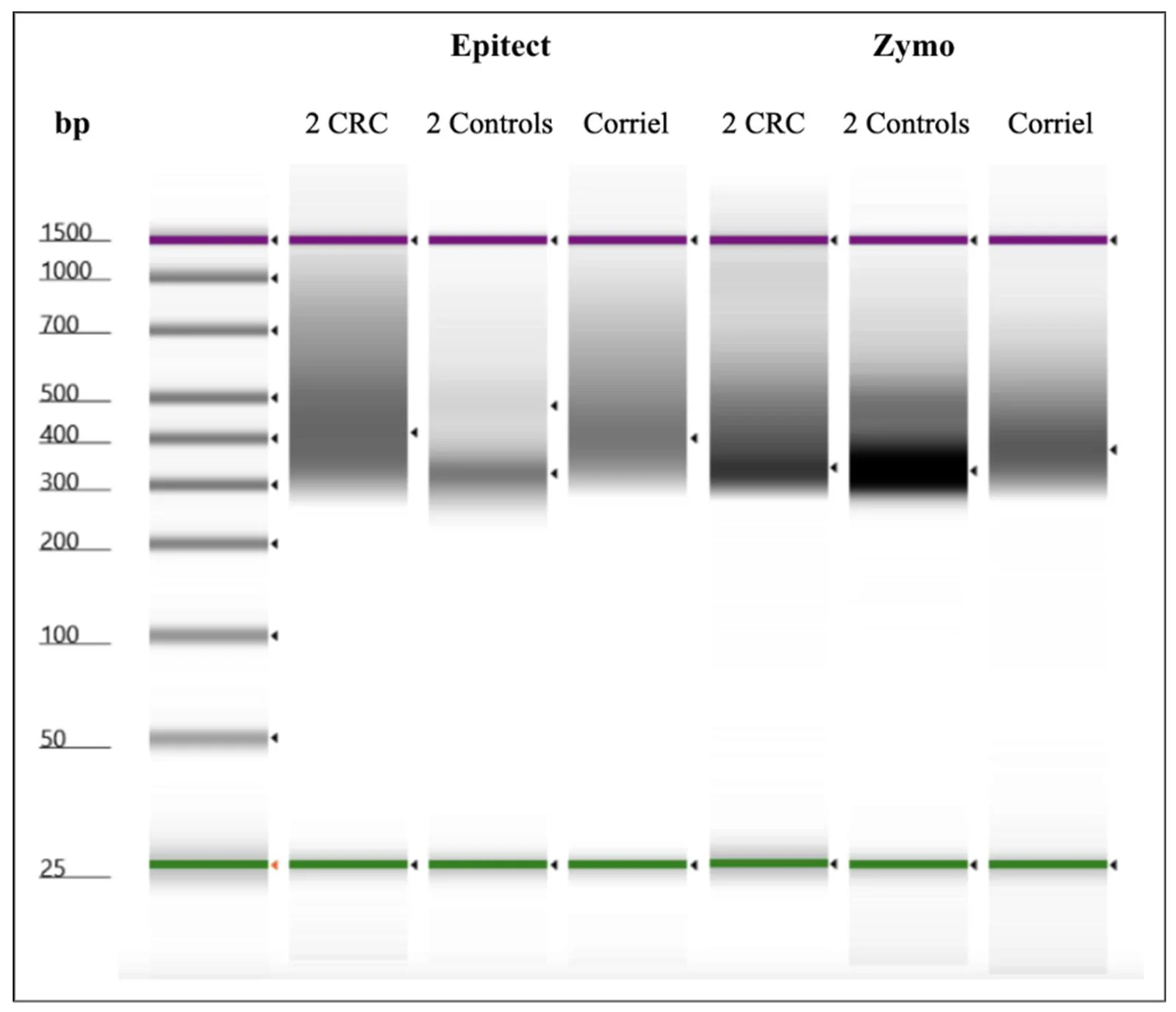
TapeStation profiling of cfDNA libraries across analyzed samples (4 cases and 1 Corriel-NA12878-N88 reference sample).

**Figure 3:**
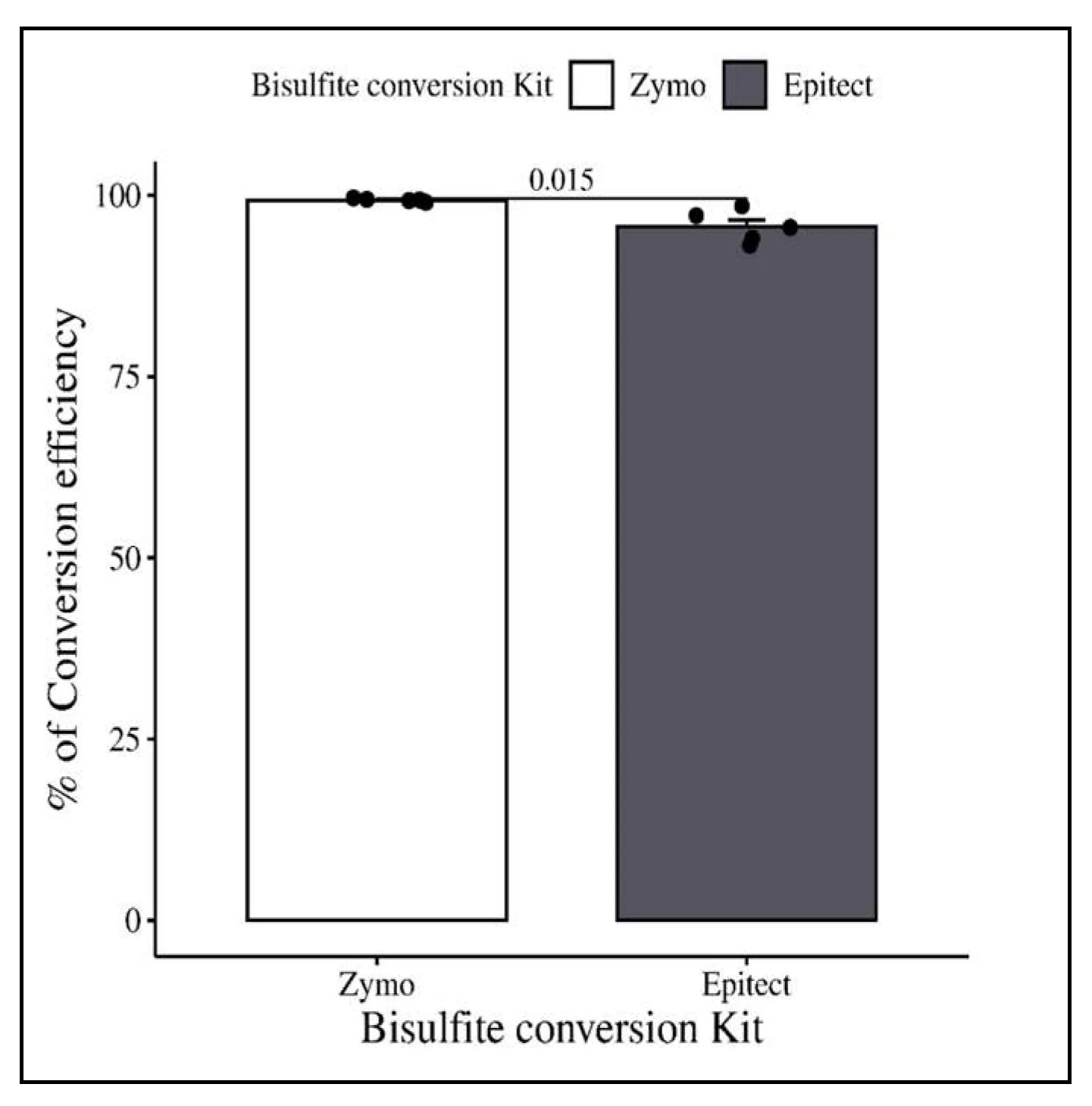
Comparison of conversion efficiency between Zymo and EpiTect Bisulfite Conversion Kits. Data are presented as individual paired observations together with the mean ± SD (n = 5). Lines connecting measurements represent the same biological sample processed using the two conversion protocols. The mean conversion efficiency was 99.30 ± 0.00% for Zymo and 95.70 ± 1.98% for EpiTect. The mean paired difference was 3.60% (95% CI: 1.14–6.06%). The conversion efficiency was significantly higher for Zymo than for EpiTect (paired *t*-test, *p* = 0.0154).

**Figure 4:**
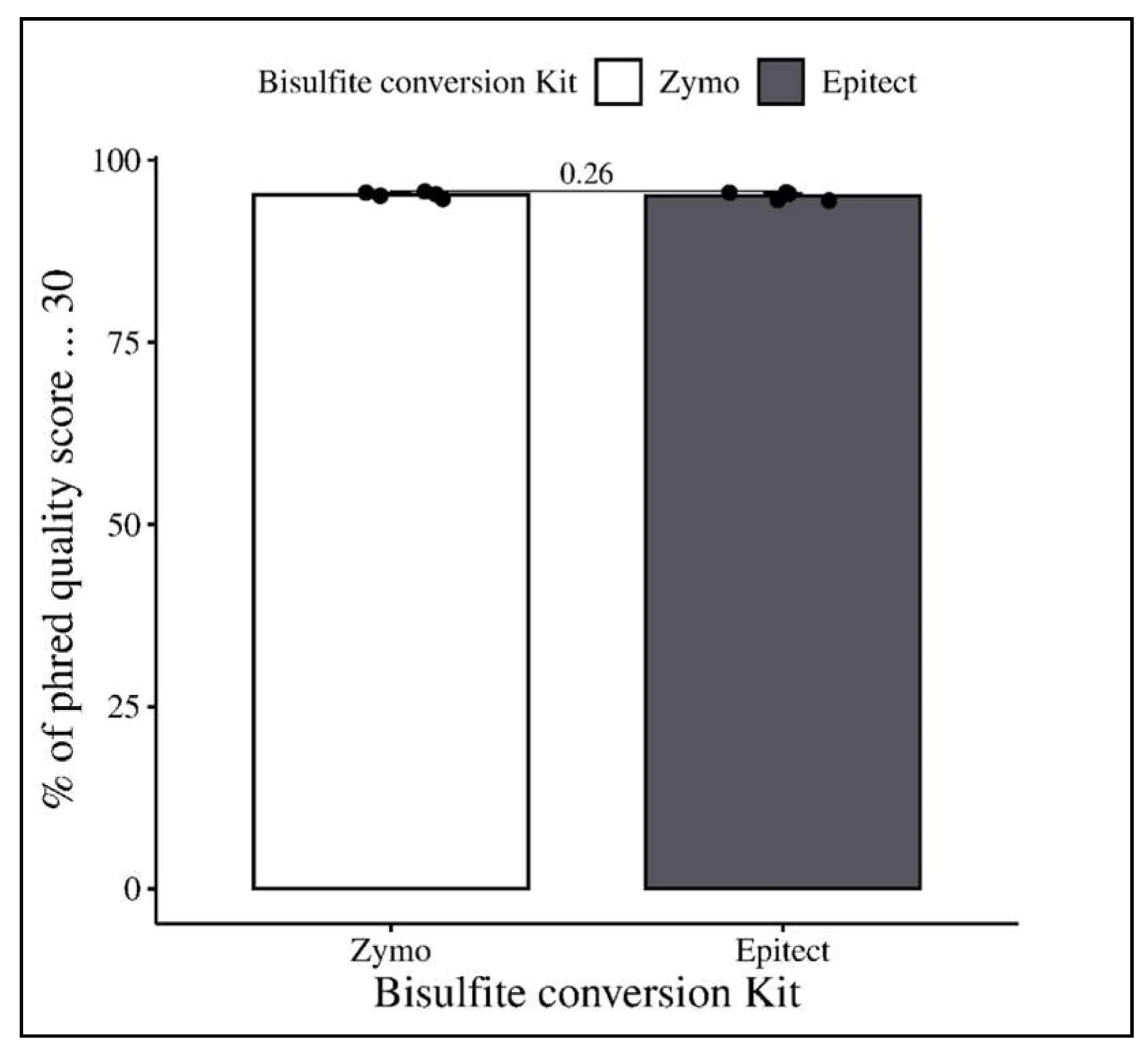
Comparison of percentage of sequenced bases with a phred quality score ≥ 30 between Zymo and EpiTect Kits. Data are presented as individual paired observations together with the mean ± SD (n = 5). Lines connecting measurements represent the same biological sample processed with the two conversion protocols. The mean Q30 percentage was 95.26 ± 0.39% for Zymo and 95.10 ± 0.55% for EpiTect. The mean paired difference was 0.16 percentage points (95% CI: −0.18–0.50). The difference in Q30 percentage between the two conversion protocols was not statistically significant (paired *t*-test, *p* = 0.2560).

**Figure 5:**
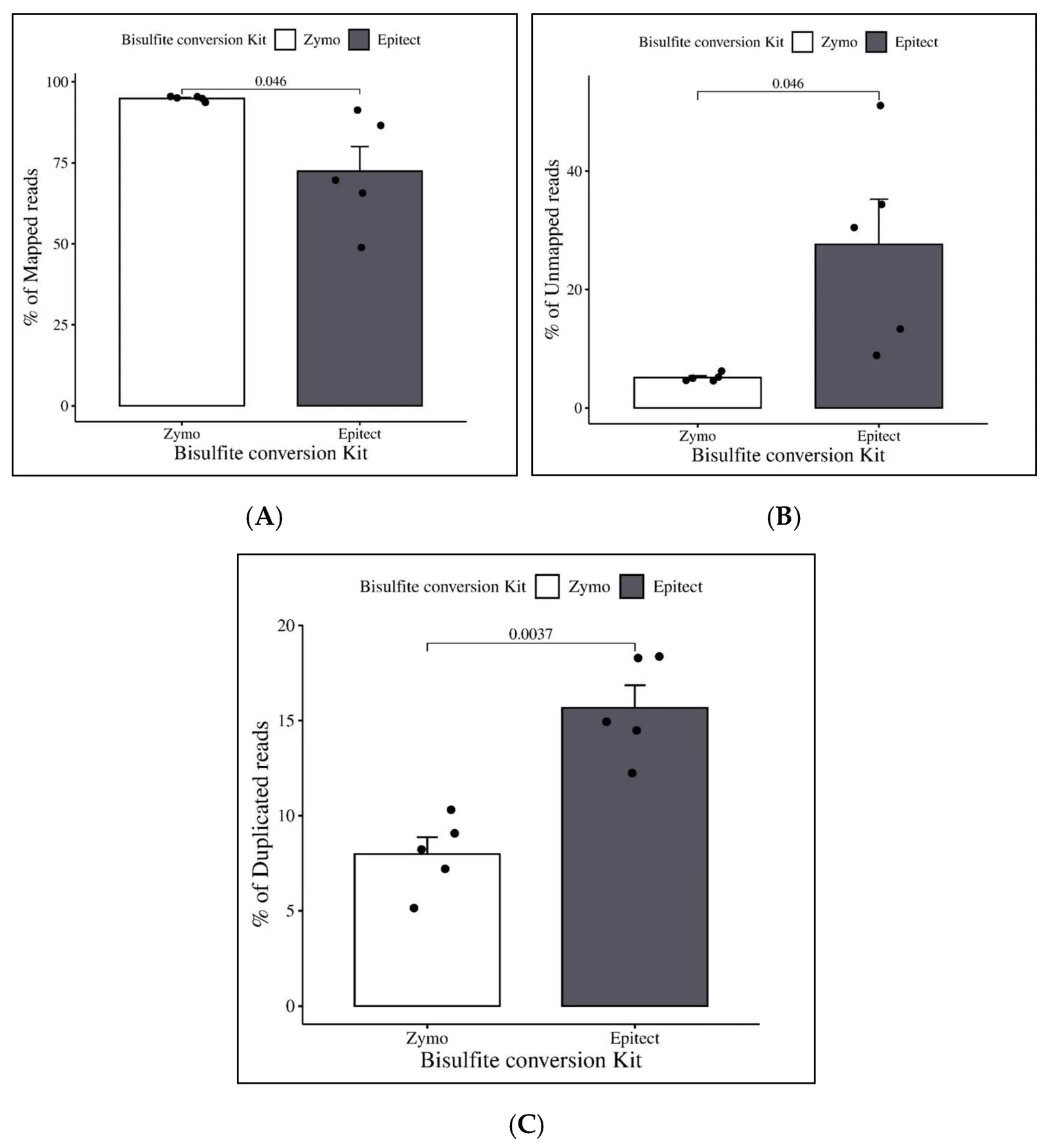
Comparison of mapping metrics between Zymo and EpiTect kits. Data are presented as individual paired observations with mean ± SD (n = 5). (**A**) Zymo showed a higher mapping rate than EpiTect (94.86 ± 0.70% vs. 72.40 ± 17.03%; *p* = 0.0457). (**B**) Zymo had a lower percentage of unmapped reads (5.14 ± 0.70% vs. 27.62 ± 17.04%; *p* = 0.0457). (**C**) Zymo also exhibited a lower duplication rate than EpiTect (7.98 ± 1.97% vs. 15.66 ± 2.66%; *p* = 0.0037). All differences were statistically significant based on paired *t*-tests.

**Figure 6:**
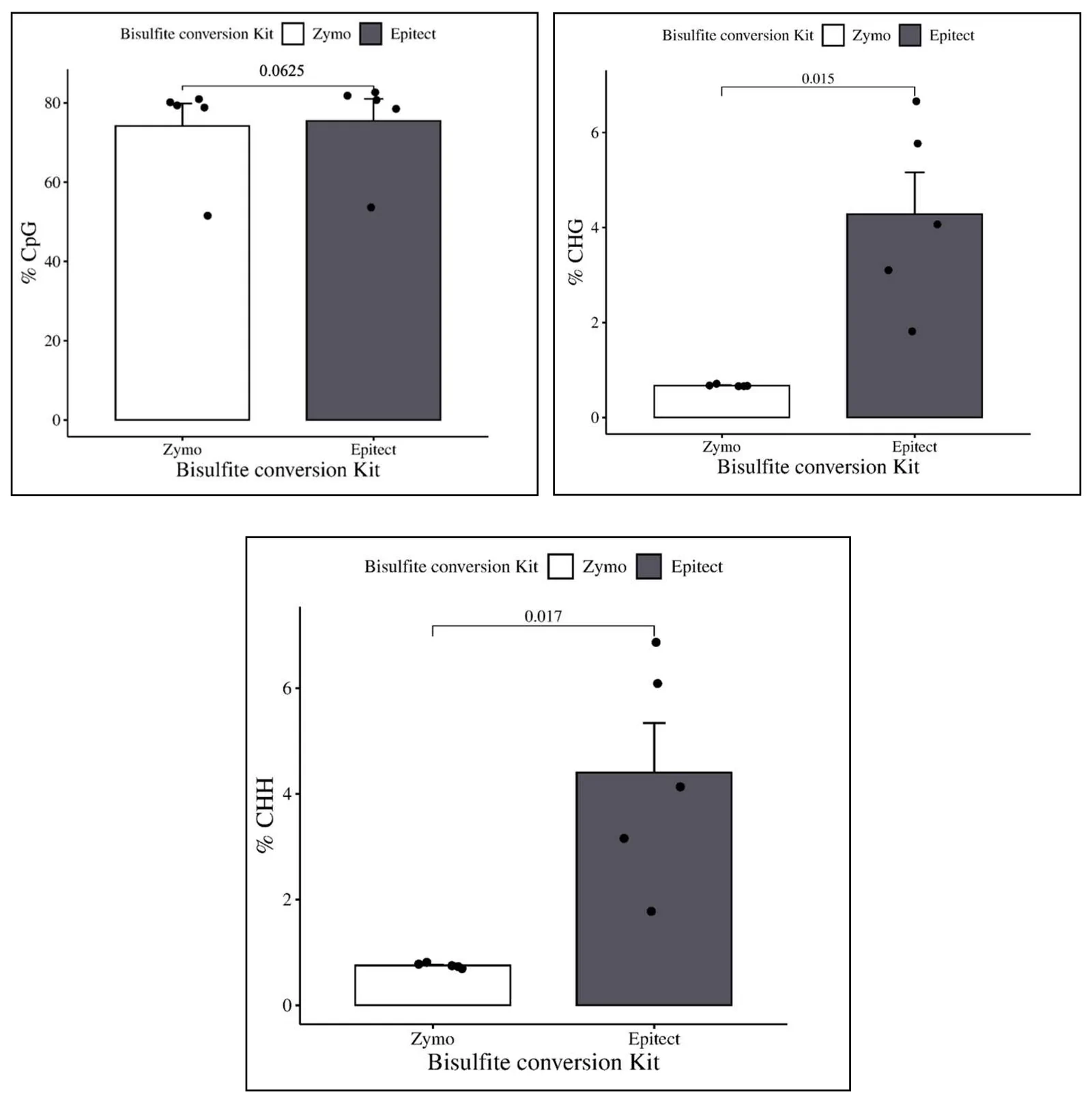
Comparison of DNA methylation levels across CpG, CHG, and CHH contexts between the Zymo and EpiTect conversion kits. Data are presented as individual paired observations with mean ± SD (n = 5). CpG methylation levels were similar between Zymo and EpiTect (74.17 ± 12.64% vs. 75.47 ± 12.35%; Wilcoxon signed-rank test, *p* = 0.0625), with no statistically significant difference. CHG methylation was significantly lower with Zymo than with EpiTect (0.674 ± 0.021% vs. 4.282 ± 1.964%; *p* = 0.0149). CHH methylation was also significantly lower with Zymo than with EpiTect (0.752 ± 0.041% vs. 4.404 ± 2.092%; *p* = 0.0173). Shapiro–Wilk and Paired Student’s *t*-tests were used for normally distributed comparisons, whereas the paired Wilcoxon signed-rank test was used for the non-normally distributed CpG methylation data.

**Figure 7:**
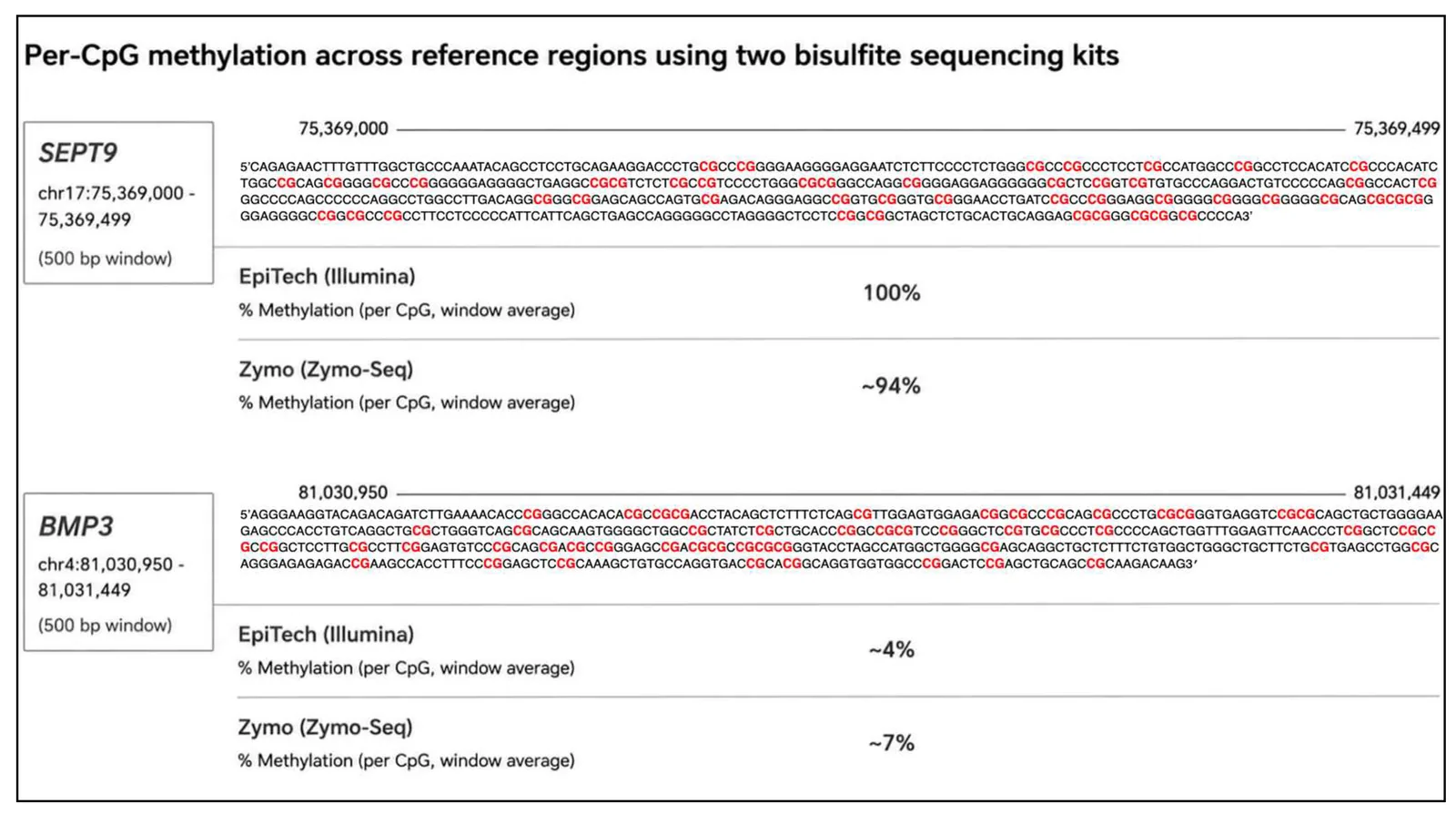
Comparison of Bismark-derived methylation levels between Zymo and EpiTect protocols across SEPT9 and BMP3 reference regions.

## Data Availability

The data that support the findings of this study are available on request from the corresponding author (Hicham Mansour: h.mansour@ump.ac.ma) and co-corresponding author (Scott Kopetz: skopetz@mdanderson.org). The data are not publicly available due to restrictions.

## Supplementary Materials

The following supporting information can be downloaded at: https://www.mdpi.com/article/10.3390/cancers18193148/s1, Table S1: DNA Yield After Extraction and DNA Input for Bisulfite Conversion and Final Library Quantification Using Qubit and qPCR; Table S2: Comparison of sequencing library quality, mapping performance, duplication rate, and DNA methylation profiles between Zymo and EpiTect bisulfite conversion methods; Table S3: TapeStation-Based Assessment of DNA Library Integrity and Quality in CRC, Control, and Corriel Samples.
