# Supplementary material for: A Pilot Head-to-Head Comparison of Two Bisulfite Conversion Kits for NGS-Based DNA Methylation Analysis of Liquid Biopsy Samples from Colorectal Cancer
Source: Cancers (Basel). 2026 Sep 29;18(19):3148. doi: 10.3390/cancers18193148 (PMC13664151; doi:10.3390/cancers18193148)
Supplement: Supplementary file 1 [file cancers-18-03148-s001.zip › cancers-4468379-supplementary.pdf]

Supplementary materials

Table S1 : DNA Yield After Extraction and DNA Input for Bisulfite Conversion and Final Library Quantification Using Qubit and qPCR

Sample	DNA concentration after extraction Qubit (ng/µl)	Total DNA used for Bisulfite Converstion (ng)	Converted Elution volume (µl)	Final Library Qubit (ng/µl)	Final Library QPCR (nM)
CRC 1 - EpiTect	3,5	21	20	3,66	26,24582536
CRC 2 - EpiTect	2,18	13,08	20	3,63	23,38237879
Control 1 - EpiTect	26,4	165	20	10,5	64,26684429
Control 2 - EpiTect	2,09	11,495	20	3,36	22,46928996
Corriel - EpiTect	378	400	20	12,6	91,66681393
CRC 1- Zymo	3,5	21	20	12,2	145,1811241
CRC 2 - Zymo	2,18	13,08	20	14,4	139,8121618
Control 1 -Zymo	26,4	165	20	12,5	133,4935871
Control 2 - Zymo	2,09	11,495	20	10,6	89,91368337
Corriel - Zymo	378	400	20	12,8	118,3143984

Table S2: Comparison of sequencing library quality, mapping performance, duplication rate, and DNA methylation profiles between Zymo and EpiTect bisulfite conversion methods

Metric	Zymo mean ± SD	EpiTect mean ± SD	Mean paired difference	95% CI for difference	Paired *p*-value
Library concentration by qPCR (nM)	125.34 ± 22.22	45.61 ± 31.12	79.73	31.79–127.68	0.0099
Library concentration by Qubit (ng/µl)	12.50 ± 1.36	6.75 ± 4.45	5.75	0.19–11.31	0.0453
TapeStation concentration (pg/µL)	4153.33 ± 1827.63	1309.67 ± 511.65	2843.67	−480.92–6168.26	0.0665
Conversion efficiency (%)	99.30 ± 0.00	95.70 ± 1.98	3.60	1.14–6.06	0.0154
Q30 (%)	95.26 ± 0.39	95.10 ± 0.55	0.16	−0.18–0.50	0.2560
Mapping rate (%)	94.86 ± 0.70	72.40 ± 17.03	22.46	0.69–44.23	0.0457
Unmapped reads (%)	5.14 ± 0.70	27.62 ± 17.04	-22.48	−44.26–−0.70	0.0457
Duplication rate (%)	7.98 ± 1.97	15.66 ± 2.66	-7.68	−11.20–−4.16	0.0037

CpG methylation (%)	74.17 ± 12.64	75.47 ± 12.35	-1.31	−2.75–0.14	0.0665
CHG methylation (%)	0.674 ± 0.021	4.282 ± 1.964	-3.61	−6.05–−1.16	0.0149
CHH methylation (%)	0.752 ± 0.041	4.404 ± 2.092	-3.65	−6.24–−1.06	0.0173

Table S3: TapeStation-Based Assessment of DNA Library Integrity and Quality in CRC, Control, and Corriel Samples

Samples	Size [bp]	Calibrated Conc. [pg/µl]	Peak Molarity [pmol/l]	% Integrated Area
2 CRC (EpiTect)	415	819	3040	100
2 Controls (EpiTect)	325	1400	6650	76.30
	480	436	1400	23.70
Corriel (EpiTect)	400	1270	4900	100
2 CRC (Zymo)	336	2750	12600	100
2 Controls (Zymo)	330	6220	29000	100
Corriel (Zymo)	376	3490	14300	100
